# Aspirin Alleviates Particulate Matter Induced Asymptomatic Orchitis of Mice *via* Suppression of cGAS-STING Signaling

**DOI:** 10.3389/fimmu.2021.734546

**Published:** 2021-12-01

**Authors:** Tengyu Zhu, Xue Chen, Huan Qiu, Yang Liu, James Mwangi, Ling Zhao, Wenjun Ding, Ren Lai, Lin Jin

**Affiliations:** ^1^ Key Laboratory of Animal Models and Human Disease Mechanisms of Chinese Academy of Sciences/Key Laboratory of Bioactive Peptides of Yunnan Province, Kunming Institute of Zoology, Chinese Academy of Sciences, Kunming, China; ^2^ Kunming College of Life Science, University of Chinese Academy of Sciences, Kunming, China; ^3^ College of Life Sciences, Nanjing Agricultural University, Nanjing, China; ^4^ Experimental Animal Center, Kunming Institute of Zoology, Chinese Academy of Sciences, Kunming, China; ^5^ Laboratory of Environment and Health, University of Chinese Academy of Sciences, Beijing, China; ^6^ Kunming institute of zoology-the Chinese university of Hong Kong (KIZ-CUHK) Joint Laboratory of Bioresources and Molecular Research in Common Diseases, Kunming Institute of Zoology, Chinese Academy of Sciences, Kunming, China

**Keywords:** male infertility, particulate matter, orchitis, aspirin, cGAS-STING pathway

## Abstract

As an important source of air pollutant, airborne particulate matter (PM) has become a major threat to public health. Orchitis is characterized by acute or chronic testicular inflammation and is a primary cause of male infertility. Although accumulating evidence indicates that PM exposure is associated with increased male infertility rates, the mechanism by which PM is involved is not well understood. Here, we found that short-term PM exposure activated NF-κB signaling in mouse Leydig cells and testes and leading to asymptomatic orchitis. Analyzing the mitochondrial abundance and cGAMP levels in PM exposed mouse Leydig cells, we found that PM exposure induced mitochondrial injury and mtDNA release, leading to inflammation *via* the cGAS-STING axis. We also found that aspirin-induced acetylation of cGAS inhibited the inflammation in mice after PM exposure, especially in the testes. Moreover, aspirin pretreatment rescued offspring growth in PM-exposed mice. In summary, our study not only provides evidence that PM-induced asymptomatic orchitis in mice may be amenable to aspirin pre-treatment by acetylating cGAS, but also provides a potential explanation for male infertility caused by air pollutants.

## Introduction

With the rapid industrial development, ambient air pollution has become a major threat to public health in China. As an important source of air pollutant, airborne particulate matter (PM) is a complex mixture of inorganic and organic components (1). Of note, exposure to PM with a mean diameter of less than 10 μm (PM_10_ and PM_2.5_) can lead to multiple system damage (2), including adverse impacts on the cardiovascular and respiratory systems. Inhalation of PM_2.5_ can lead to oxidative stress in the respiratory tract and may increase the risk of lung cancer and chronic airway inflammatory diseases (3, 4). Furthermore, It is reported that PM2.5 exposure can induces apoptosis and autophagy in human lung epithelial cells (5). Of concern, exposure to PM enhanced ACE2 and TMPRSS2 expression and enhance the SARS-CoV-2 susceptibility *in vivo* (6, 7). PM exposure is not only associated with human endothelial injury and cardiovascular diseases, but also with cardiac dysfunction in mice offspring (8, 9). As such, considerably more efforts should be expended understand the health impact of PM exposure.

The estimated prevalence of infertility among reproductive-aged couples in China has increased to 25% in recent years (10). Several studies have indicated that PM exposure may be associated with low sperm quality and increased infertility rates in males (11, 12). About 70% of male infertility cases are caused by abnormal spermatogenesis (13). Instillation of PM is known to lower sperm counts and increased sperm morphology rate in rats *via* PI3K/Akt signaling (14). Moreover, exposure to concentrated ambient PM_2.5_ can impair spermatogenesis in mice (15). Immune homeostasis in mammalian testes is essential for testicular function. Disruption of testicular immunoregulation can lead to orchitis and male infertility (16). However, although immunological factors are important in spermatogenesis and may contribute to PM-induced male infertility, they remain poorly understood (16, 17).

In the present study, we examined the adverse impacts of PM exposure *in vitro* and *in vivo* on the male reproductive system in mice. Results showed that exposure to PM led to asymptomatic but not acute orchitis *via* cyclic guanosine monophosphate-adenosine monophosphate (GMP-AMP) synthase (cGAS)-stimulator of interferon gene (STING)-mediated NF-κB signaling. cGAS is a cytosolic DNA sensor which induces type I IFN (IFN-I) by producing the endogenous second messenger cyclic GMP-AMP (cGAMP) (18). cGAMP is a secondary messenger that binds and activates STING (stimulator of interferon genes, also known as TMEM173, MITA, ERIS and MPYS) on the endoplasmic reticulum (ER) membrane (19–21). The broad roles of cGAS-STING and NF-κB signaling in immunoregulation suggest that the inhibitory agents may be applicable for rebalancing the immune homeostasis (22–24). Therefore, we explored the potential molecular mechanisms involved in PM-induced disruption of the testicular immune homeostasis and abnormal spermatogenesis. In addition, we also explored the possible solutions that may alleviate the PM-induced asymptomatic orchitis.

## Materials and Methods

### Cell Lines and Mice

TM3 cells (mouse Leydig cell line) were obtained from the Kunming Cell Bank, Kunming Institute of Zoology, Chinese Academy of Science. TM3 cells were cultured in Dulbecco’s Modified Eagle Medium and Ham’s F-12 medium (DMEM/F12) (Gibco, USA) supplemented with 5% fetal bovine serum (FBS) and, 2.5% horse serum, 100 U/ml penicillin and 100 μg/ml streptomycin in 5% CO_2_ at 37°C. Male 6- to 8-week-old male C57/B6 mice were purchased from the Animal Resource Center at the Kunming Institute of Zoology. Institutional Review Board approval was obtained from our institute for this study. All mice were kept under specific pathogen-free conditions and all animal experiments were conducted in accordance with the guidelines and were approval of the Kunming Institute of Zoology, Chinese Academy of Sciences Animal Care and Use Committee (SMKX-2019031).

### PM Exposure in Mice

Urban particulate matter, PM (1648a, NIST, USA), lLipopolysaccharides from *Escherichia coli* O111:B4 (L2630, Sigma, USA) and Pyrrolidinedithiocarbamate ammonium, PDTC (PDTC, T3147, TargetMol, China) and aspirin (HY-14654, MCE, China) were used in this study. PM was suspended in PBS to make a final concentration of 10 mg/ml. 6- to 8-week-old male C57/B6 mice were intranasally inoculated with 400 μg of PM (40 μl) every day for 7 days. For drugs administration, mice were intraperitoneally injected with PDTC (30 mg/kg) or intragastrically administered with aspirin (20 mg/kg/day) 1 hour before PM exposure according to the instructions and established studies (25–27).

### Preparation of Tissue Samples for Hematoxylin and Eosin (H&E) Staining

The lungs of mice were fixed in 10% formalin and the left side of testes were fixed in Bouin fix solution (SL1590, Coolaber, China). After fixation, 5-μm tissue sections were prepared and stained with H&E and examined.

### Flow Cytometry

Single-cell suspensions of the lung samples were prepared by mechanical pressing and filtering through 70-mm cell strainers. Cells were stained as per our previous study (28) and the following antibodies were used. Mouse Integrin alpha M/CD11b Alexa Fluor^®^ 405-conjugated antibody (FAB1124V, abcam); anti-mouse LY-6G (RB6-8C5)-APC (17-5931-81, eBioscience); and ICAM-1 monoclonal antibody (YN1/1.7.4)-Pacific Blue (A15404, eBioscience). Fluorescence Activated Cell Sorter (FACS) analysis was performed on a BD FACS Calibur flow cytometer. Data analysis was performed using Flowjo v10 software.

### *In Vitro* Exposure to PM

For PM exposure, the TM3 cells in free antibiotics medium were seeded into 12-well plates at ~1 × 10^6^ cells/well. Cells were exposed to PM with or without PDTC, and aspirin administration for indicated times. Cells were lysed in Radio-Immunoprecipitation Assay buffer (RIPA buffer) with protease (HY-K0010, MCE) and phosphatase inhibitors (HY-K0022, MCE) for immunoblot analysis and lysed in TRIzol reagent (Invitrogen) for RNA isolation.

### Gene Knockdown Assays

TM3 cells were transiently transfected with pGFP-C-shLenti vector containing cGAS shRNA (GTTCAATCTATTCTCTCAAGAACTAATTG) or p65 shRNA (#1 GCATGCGATTCCGCTATAAAT, #2 GGAGTACCCTGAAGCTATAAC, #3 AGCGAATCCAGACCAACAATA) with lipofectamine 3000 transfection reagent (Thermo Fisher Scientific) according to manufacturer’s protocol. The transfected cells were incubated for 48–72 h at 37°C and the knockdown efficiency was analyzed by western blot before further experiments.

### Quantitative Real-Time Polymerase Chain Reaction (qRT-PCR) and RNA Sequencing (RNA-Seq)

Total RNA was isolated from cells and tissues by using TRIzol reagent (Invitrogen). cDNA was reverse-transcribed by using M-MLV reverse transcriptase (M1705, Promega, USA). Real-time qRT-PCR was performed on the StepOnePlus Real-Time PCR Systems (Thermo, USA). All primer sequences are listed in **Supplementary Table 1**.

RNA-seq and data analysis were performed by a commercial service (Novogene, China). A log_2_ fold-change of 1 was set as the threshold for significantly differential expression. The heatmap was generated by using GraphPad v8.0 software to show the normalized gene expression.

### Immunoblot Analysis

Nuclear protein was isolated using a NE-PER™ Nuclear and Cytoplasmic Extraction kit (78833, Thermo Fisher Scientific) according to the manufacturer’s manual. Total proteins were extracted from cells or tissues for western blotting analysis. Primary antibodies included: anti-cGAS (sc-515803, Santa Cruz), anti-ICAM1 (ab179707, Abcam), anti-VCAM1 (ab134047, Abcam), anti-p-IκBα (2859L, CST), anti-IκBα (9242L, CST), anti-p-p65 (3033, CST), anti-p65 (8242, CST), anti-lamin B1 (13435S, CST), anti-GAPDH (T0004-50, Affinity Biosciences) and anti-β-actin (sc-81178, Santa Cruz). Secondary antibodies included horseradish peroxidase (HRP)-labeled anti-rabbit, anti-mouse, and anti-goat antibodies (KPL). Total proteins were separated by 12% sodium dodecyl sulfate polyacrylamide gel electrophoresis (SDS-PAGE) and electro-transferred onto a polyvinylidenedifluoride (PVDF) membranes (Roche, Germany). The PVDF membranes were blocked with TBST (2.42 g/L Tris base, 8 g/L NaCl, 0.1% Tween 20, pH 7.6) containing 5% non-fat dried milk (BD, USA) at room temperature for 2 h. After washing three times with TBST, the membrane was incubated overnight with primary antibodies at 4°C, then incubated with the secondary antibodies for 1 h at room temperature. After washing with TBST, the membrane were developed with an enhanced chemiluminescence kit (TIANGEN, China) on an ImageQuant LAS 4000 mini system (GE Healthcare Life Sciences).

### Sperm Morphology, Motility and Sperm Counts

Sperm were collected from the cauda epididymides of mice. Sperm morphology analysis and sperm count were determined according to previous studies (14, 15). Abnormal sperm morphology rates were calculated by counting all sperms with abnormal heads or tails. Sperm motility was analyzed using a computer-aided sperm analysis system with Hamilton Thorne IVOS II (Beverly, MA, USA).

### Immunofluorescence Staining and Microscopy

Cells were fixed in 4% paraformaldehyde for 15 min at room temperature and were then permeabilized with 0.3% Triton X-100 (T0694, Amresco) for 20 min, washed with PBS, and blocked in 2% BSA for 1 h. Cells were stained with antibodies overnight at 4°C. After washing, cells were stained with a fluorescence-conjugated secondary antibody with FITC-Phallodidin (40735ES75, Yeasen) for 30 min, and mounted using ProLong Gold Antifade Reagent with DAPI (8961S, CST).

For cryosections, tissues were harvested and fixed as mentioned above. The 5-μm sections were washed with PBS and blocked with the blocking buffer. The sections were stained with primary antibodies anti-CD11c (AP-MAB0814) and anti-F4/80 (ab6640, Abcam) at 4°C overnight. After washing, slides were stained with secondary antibodies for 30 min, and mounted using ProLong Gold Antifade Reagent with DAPI (8961S, CST). Immunostaining was detected with an Olympus FluoView 1000 confocal microscope (Olympus, Melville, NY, USA). The intensity of signaling was quantified by ImageJ 1.8.0 software (National Institutes of Health, USA).

### Mitochondria Abundance and ATP Levels

Mitochondria abundance was analyzed according to the previous study (29). ATP levels were determined by using a ATP detection kit according to the instruction (S0027, Beyotime, China).

### cGAMP Extraction and Quantification Using LC–MS/MRM

The cGAMP extraction from cells was carried out according to the previous research (30). After PM stimulation, the cells were washed with PBS and lysed with 1 mL of cold extraction solvent (40:40:20 (v/v/v) methanol-acetonitrile-water). The cell lysates were stored at -20°C for 1 hour and then centrifuged at 16,000×g for 5 min. The supernatants were lyophilized on the lyophilizer and the pellets were resuspended in 200 μL of ammonium acetate buffer (10 mM ammonium acetate, 0.05% acetate in water). After centrifugation at 16,000×g for 10 min, the supernatants were quantified by liquid chromatography-MS/multiple reaction monitoring (LC-MS/MRM).

The LC-MS/MS system consisted of an Acquity H-Class UPLC system, coupled to a Xevo TQ-S Micro Tandem Mass Spectrometer (Waters, Manchester, UK). Chromatographic separation was performed by injecting 10 μL of supernatant onto a Waters Acquity UPLC HSS T3 column (2.1 × 50 mm, 1.8 μm particle size, Waters). The mass spectrometer was operated in electrospray positive ionization mode, with capillary was maintained at 2.5 kV and the source temperature of 150°C The desolvation gas flow and temperature were 600 L/h and 350°C, respectively. The cone voltage was 60 V for all analytes with a source offset of 30 V. Transitions were monitored in multiple reaction monitoring (MRM) mode with a dwell time of 0.091 s.

### Statistical Analysis

Two-way analysis of variance (ANOVA) and Fisher’s least significant difference (LSD) tests were used to analyze the bodyweight changes in mice. Statistical analysis was performed using two-tailed Student *t*-test and log-rank tests where applicable. A *P*-value ≤ 0.05 was considered significant. All data analyses were performed using GraphPad Prism 6 (GraphPad Software, USA).

## Results

### Short-Term Exposure to PM Causes Inflammation in Lungs and Testes of Mice

**Figure 1:**
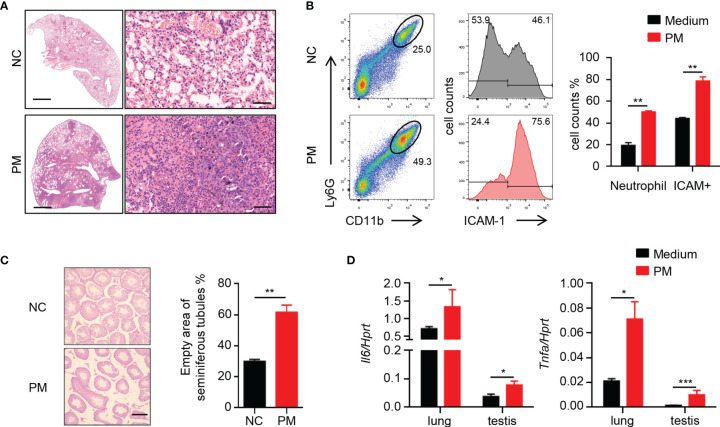
Short-term PM exposure causes inflammation in lungs and testes of mice. **(A)** Representative H&E staining images show the inflammatory cell infiltration in lung after 7 days of PM exposure. Scale bars: 2000 μm on left, 50 μm on right. **(B)** FACS analysis of neutrophils and ICAM-1^+^ cells in lungs of mice after 7 days of PM exposure. The percentage of total cells was quantified (right). **(C)** H&E staining evaluation of empty area of seminiferous tubules in testes of mice after 7 days of PM exposure. The percentage of the empty area was quantified (right). Scale bars: 50 μm. **(D)** Expression analysis of *Il6* and *Tnfa* in lung and testes after 7 days of PM exposure. There were 6 mice in each group. Data represent two independent experiments. Data are mean ± standard error of the mean (SEM). *P < 0.05; **P < 0.01; ***P < 0.001.

A broad consensus is that inhaled PM with small particle size can enter the blood circulation and then consequently affect other organs. To determine the effects of PM on mice, 8-week-old male mice were exposed to 400 μg of PM per day. After 7 days, the PM-exposed mice developed severe pneumonia-like symptoms. Inflammatory cells in the lungs of mice were significantly increased after PM exposure (**Figure 1A**). FACS analysis showed that the percentages of neutrophils and ICAM-1^+^ cells in the lungs of mice were also increased after PM exposure (**Figure 1B**). Furthermore, inflammation of other tissues was observed (**Supplementary Figure 1**). In the testes, the empty areas of seminiferous tubules were remarkably enlarged after PM exposure (**Figure 1C**). This type of lesion often occurs when the testis of mice become senile, inflamed or injured. Therefore, we examined the expression levels of certain inflammatory cytokines in the lungs and testes of these mice. As shown in **Figure 1D**, the expression levels of *Il6* and *Tnfa* in the lungs and testes sample were up-regulated after PM exposure. These observations suggested that PM exposure in not only causes pulmonary inflammation, but also inflammatory injury of the testes.

### PM Activates NF-κB Signaling and Induces Asymptomatic Orchitis in Mice

**Figure 2:**
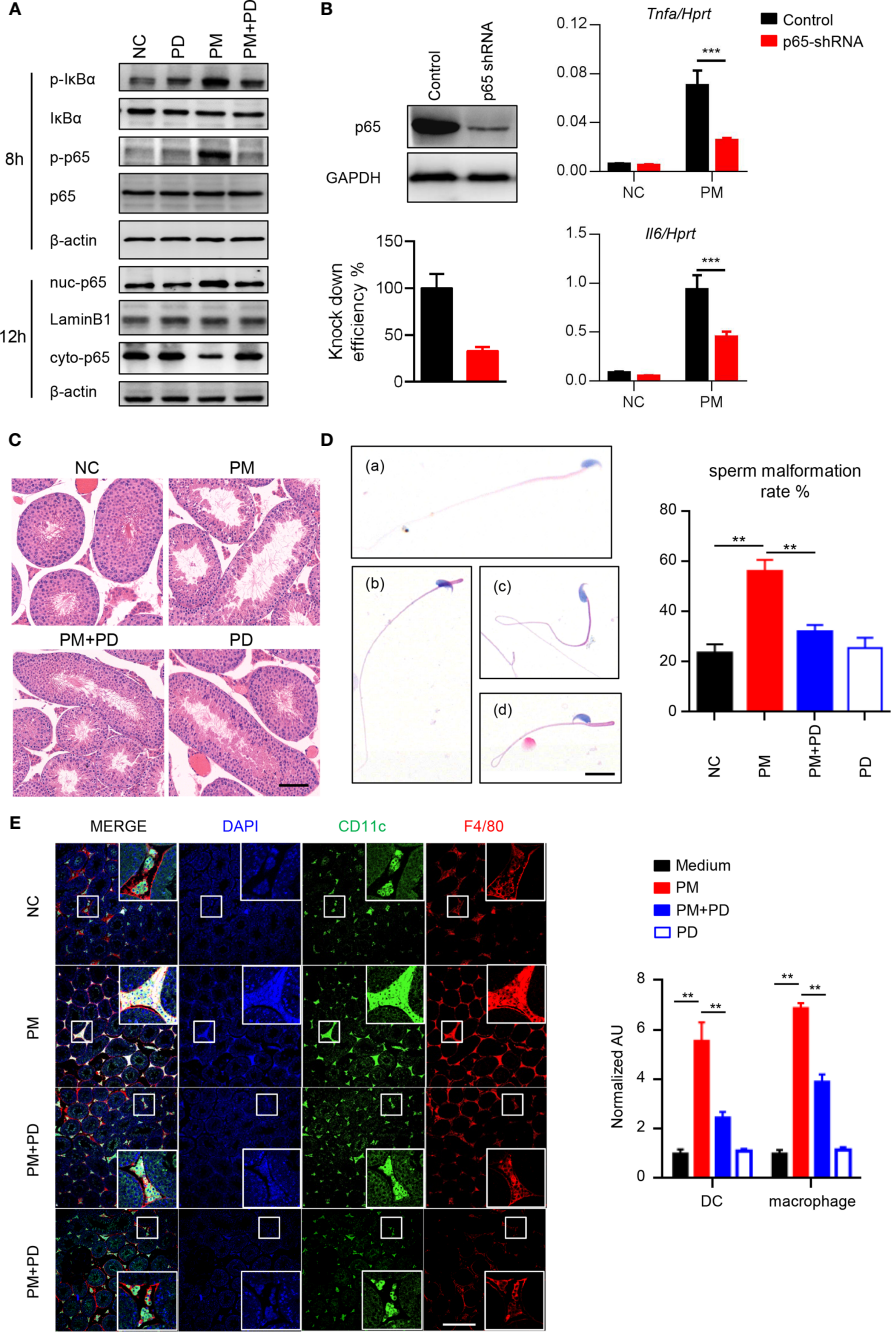
PM exposure induced orchitis in mice is NF-κB signaling dependent. **(A)** Immunoblot analysis of phosphorylation of IκBα and p65 and protein level of IκBα and p65 in TM3 cells after 200 μg/ml PM administration for 8 hours. β-actin was used as the loading control. **(B)** Expression analysis of *Il6* and *Tnfa* in the p65 knockdown TM3 cells after 200 μg/ml PM administration for 12 hours. The knockdown efficiency was analyzed by western blot 48 h post transfection (left panel). The intensity of signaling was quantified. **(C)** H&E staining evaluation of empty areas of seminiferous tubules in testes of mice after 7 days of PM exposure with or without PDTC administration. In **(C–E)**, NC, PBS; PM, 400 μg/day PM; PM+PD, 400 μg/day PM + PDTC; PD, PDTC only. Scale bar: 20 μm. **(D)** H&E staining of typical abnormal sperm morphology: (a) normal sperm; (b) abnormal sperm head; (c) abnormal sperm tail; (d) abnormal sperm head and tail. Scale bars: 10 μm. Sperm malformation rate was quantified (right). **(E)** Confocal immunofluorescence analysis of dendritic cells and macrophages in cryosections of testes of mice after 7 days of PM exposure with or without PDTC administration. Scale bars: 100 μm. There were 5-6 mice in each group. Data represent three independent experiments in **(A)** and two independent experiments in **(B–E)**. Data are presented as mean ± SEM. **P < 0.01; ***P < 0.001.

To determine the role of PM in testicular inflammation, we performed genome-wide expression analysis to profile differentially expressed genes in mouse Leydig cells with or without PM administration. Among the genes significantly upregulated after PM treatment, most were involved in NF-κB signaling (**Supplementary Figure 2A**). This finding suggests that PM may induce testis inflammation *via* activation of NF-κB signaling in mouse Leydig cells. As predicted, levels of phosphorylated IκBα and p65, which are critical for NF-κB signaling activation, were significantly up-regulated in the mouse Leydig cells after PM treatment (**Figure 2A**). Moreover, the nuclear localization of p65 in TM3 cells was markedly induced after PM administration (**Figure 2A**). Consistently, confocal immunofluorescence analysis also revealed that PM-induced nuclear localization of p65 in TM3 cells (**Supplemetary Figure 2B**). As NF-κB signaling regulates the expression of many inflammatory genes, the expression levels of representative genes regulated by NF-κB signaling were analyzed in the presence of LPS, PM, or NF-κB specific inhibitor (PDTC). As shown in **Supplementary Figure 2C**, the expression levels of *Mcp1*, *Il6*, *Cxcl1* and *Cxcl9* were up-regulated after PM and LPS treatment but were reduced after co-administration of PDTC. This observation was further confirmed by using p65 knockdown cells (**Figure 2B**). At 3-days post-PM exposure, the phosphorylation levels of IκBα and p65 and the levels of ICAM-1 and VCAM-1 were increased in the mouse testes (**Supplementary Figure 2D**). Notably, PDTC administration dramatically diminished the phosphorylation levels of IκBα and p65 and the levels of ICAM-1 and VCAM-1 in the testes (**Supplementary Figure 2D**). At 7 days post-PM exposure, the enlarged empty areas of seminiferous tubules were substantially reduced after PDTC administration (**Figure 2C**). An earlier study suggested that PM may impair spermatogenesis and decrease sperm counts in mice (15). Here we found an increase in abnormal sperm morphology after 7 days of PM exposure, with PM-induced sperm abnormality was altered after PDTC administration (**Figure 2D**).

Mammalian testes are immunoprivileged sites where systemic immune responses are strictly controlled (31). Considerable effort has been made to understand testicular immunoregulation in mammalian testes and recent progress has been comprehensively reviewed (16, 32). The blood-testis barrier (BTB) is formed by tight junctions between Sertoli cells near the basal lamina of the seminiferous epithelium and is important for maintaining normal testis homeostasis (16). Disruption of the BTB or immune homeostasis by stimuli can lead to immune cell invasion in the testes and impaired male fertility (16). According to the above, it is quite clear that PM exposure activates NF-κB signaling and impairs the spermatogenesis in mice. Consistently, we found that more dendritic cells and macrophages invaded the lumen of seminiferous tubules after PM exposure, while PDTC pre-administration attenuated the invasion of these cells (**Figure 2E**). Collectively, these data indicate that inhibition of NF-κB signaling by PDTC reduces inflammation and immune cell invasion in testis and may in turn attenuates PM-induced asymptomatic orchitis in mice.

### PM Exposure Induces Mitochondrial Injury and mtDNA Release to Cause Inflammation *Via* cGAS-STING *In Vitro*

**Figure 3:**
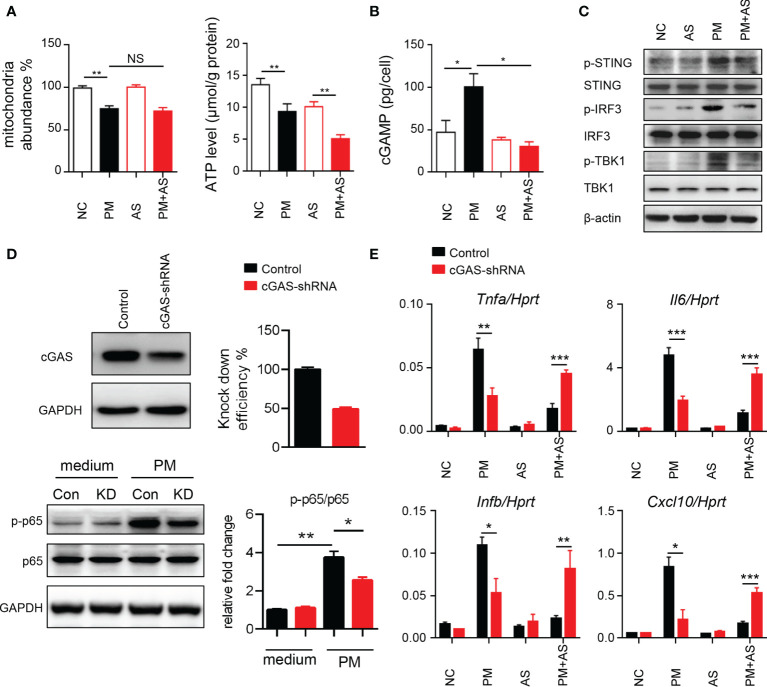
PM exposure induces mitochondrial injury and mtDNA release causing inflammation *via* cGAS-STING *in vitro*. **(A)** Relative mtDNA copy number and ATP levels from TM3 cells post 200 μg/ml PM administration for 6 hours with or without 100 μg/ml aspirin. In **(A–C)**, NC, PBS; PM, 200 μg/ml PM; AS, aspirin 100 μg/ml only; PM+AS, 200 μg/ml PM and 100 μg/ml aspirin. **(B)** Production of cGAMP from TM3 cells post 200 μg/ml PM administration for 6 hours with or without 100 μg/ml aspirin was detected by LC-MS/MRM. Data are from three technical replicates. **(C)** Immunoblot analysis of phosphorylation and protein level of STING, IRF3 and TBK1 in TM3 cells 200 μg/ml PM administration for 6 hours with or without 100 μg/ml aspirin. β-actin was used as the loading control. **(D)** Knockdown efficiency of cGAS was analyzed in TM3 cells transfected with cGAS shRNA and control shRNA at 48 hours. Immunoblot analysis of phosphorylation of p65 and protein level of p65 in cGAS knockdown TM3 cells after 200 μg/ml PM administration for 8 hours. GAPDH was used as the loading control and the intensity of signaling was quantified. Con, control shRNA; KD, cGAS shRNA. **(E)** Expression analysis of *Il6*, *Tnfa*, *Ifnb*, and *Cxcl10* in the cGAS knockdown TM3 cells after 200 μg/ml PM administration for 12 hours with or without 100 μg/ml aspirin. Data represent three independent experiments in **(A–C)** and two independent experiments in **(D, E)**. Data are presented as mean ± SEM. *P < 0.05; **P < 0.01; ***P < 0.001; NS, not significant.

To understand the mechanism underlying the activated inflammatory responses after PM exposure, we considered that exposure may induce self-mtDNA release (33). As shown in **Figure 3A**, PM administration significantly affected the mitochondria abundance and ATP levels in mouse Leydig cells. Self-DNA can trigger powerful immune responses and regulate NF-κB signaling through the cGAS-cGAMP-STING axis (34). Therefore, we analyzed cGAMP levels and signaling transduction after PM administration in mice. Aspirin was set as a control as it can directly acetylate cGAS and suppress cGAS-mediated immune responses (30). Results showed that PM indeed induced higher level of cGAMP in mouse Leydig cells (**Figure 3B**). The phosphorylation levels of STING, IRF3 and TBK1 were increased after PM treatment, but were dramatically diminished after aspirin administration (**Figure 3C**). The expression levels of *Il6*, *Tnfa*, *Ifnb* and *Cxcl10* were further analyzed post PM treatment with or without aspirin (20, 50, or 100 μg/ml) pre-treatment. As shown in **Supplementary Figure 3**, the genes up-regulated after PM exposure were suppressed by aspirin administration. Consistently, the suppression effect was significantly reduced in cGAS knockdown TM3 cells (**Figures 3D, E**). These results indicate that PM exposure induces mitochondrial injury and mtDNA release, causing inflammation *via* cGAS-STING *in vitro*, which can be reduced by aspirin pre-treatment.

### Aspirin Alleviates PM Induced Asymptomatic Orchitis in Mice and Recovers Offspring Growth

**Figure 4:**
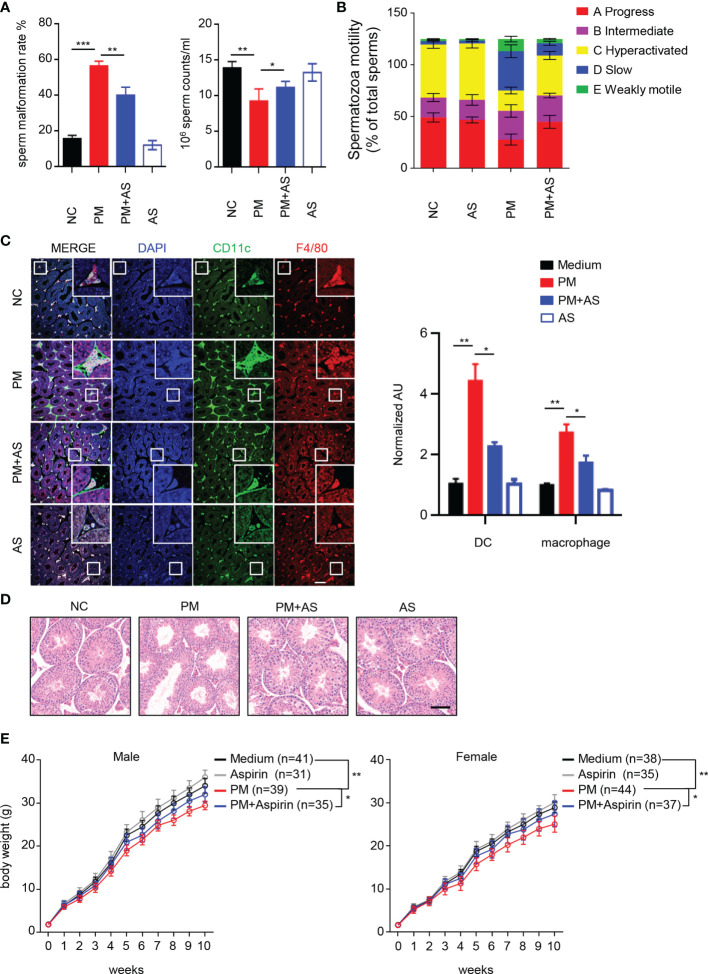
Aspirin alleviates PM-induced orchitis in mice and restores the growth of offspring. **(A)** Quantitative analysis of sperm malformation rate and sperm counts of mice (n = 6) after 7 days PM exposure with or without intragastrically administration of aspirin. In **(A–E)**: NC, PBS; PM, 400 μg/kg PM; AS, aspirin 20 mg/kg only; PM+AS, 400 μg/kg PM + 20 mg/kg aspirin. **(B)** Quantitative analysis of spermatozoa motility in mice after 7 days of PM exposure with or without aspirin administration. **(C)** Confocal immunofluorescence analysis of dendritic cells and macrophages in cryosections of mouse testes after 7 days of PM exposure with or without aspirin administration. Scale bars: 50 μm. **(D)** H&E staining evaluation of the empty area of seminiferous tubules in the testis of mice after 7 days PM exposure with or without aspirin administration. **(E)** Body weights of offspring of PM-exposed male mice. All mice were fed separately at 3 weeks of age and weighted weekly for 10 weeks. Data represent two independent experiments and are presented as mean ± SEM. *P < 0.05; **P < 0.01; ***P < 0.001.

We next investigated whether aspirin can alleviate PM-induced orchitis and other adverse effects. As shown in **Figures 4A, B**, at 7 days post exposure, intragastric administration of aspirin greatly attenuated the PM-induced sperm abnormalities and mobility reduction. Aspirin-induced acetylation of cGAS inhibited inflammation in mice after PM exposure (**Supplementary Figure 4**). In addition, we found that the protective effect of aspirin is retained for at least 3 days post stopping asipirin administration (**Supplementary Figure 5**). Consistently, the invasion of dendritic cells and macrophages in the lumen of seminiferous tubules after PM exposure was also attenuated (**Figure 4C**). Furthermore, at 7 days post exposure, the enlarged empty areas of the seminiferous tubules found after PM exposure were remarkably reduced after aspirin pre-treatment (**Figure 4D**). At 7 days post-treatment, the PM-exposed and unexposed male mice were bred with healthy females. As low quality sperm may lead to dysontogenesis of their offsprings, We further explored the consequences of PM-induced orchitis and aspirin administration on the growth and development of the mouse offsprings. Notably, the offspring of PM-exposed paternal mice grew more slowly than that of the control group (**Figure 4E**). However, this PM-induced effect on offspring was significantly reduced for those mice taking aspirin (**Figure 4E**). It is known that long term aspirin intake may have hemorrhagic complications (35). According to **Supplementary Figure 6**, we found that the hemorrhagic spot in the livers of mice emerged from day 11 post aspirin administration. Thus, these results indicate that pre-treatment of aspirin alleviates PM exposure induced orchitis in mice and rescues growth of their offspring.

## Discussion

The male reproductive system is sensitive to environmental factors such as chemical compounds and air pollutant (36, 37). As a major cause of male infertility, orchitis is characterized by acute or chronic inflammation of the testis which can be symptomatic or asymptomatic on the presentation (38, 39). Orchitis often occurs when testicular immune homeostasis is disrupted by infection or other stimuli, which can lead to male infertility (16). The acute orchitis shows significantly pathological alterations of the testis including testicular atrophy and complete loss of fertility usually caused by viruses and bacteria (40). However, despite being a major reproductive risk factor, the mechanism by which PM is involved in immune homeostasis disruption in the testes has not been well studied. As PM can enter the systemic circulation and ultimately reach the testes, both the pulmonary inflammation and the testes localized PM may break testicular immune homeostasis.

In mammals, pregnant uterus, brain and testes are typical immunoprivileged organs and their immune responses are strictly limited as they have crucial functions for the survival of a species (31, 41). Logically, testicular inflammation in the testes, approximate defined as the asymptomatic orchitis, may cause serious consequences for species reproduction. Consistent with previous studies, we found that mice exposed to PM for short time not only experienced pulmonary and systemic inflammation, but also asymptomatic orchitis (8, 15, 42). These findings suggest that PM exposure has harmful effects on testicular immune homeostasis. We also comprehensively explored genes up-regulated upon PM stimulation in mouse main testicular androgen-producing cells (TM3 cells). Leydig cells located in the seminiferous tubules of testes and are known as testicular interstitial cells. Moreover, Leydig cells can maintain spermatogenesis, control hormonal regulation, and affect secondary sexual characteristics in males (43). Of note, we found that NF-κB signaling and its specific downstream inflammatory genes were induced in TM3 cells after PM administration. This observation was further confirmed by using a NF-κB specific inhibitor and in p65 knockdown cells.

Persistent activation of NF-κB is associated with increased inflammation in the testes and with spermatogonial cell loss (38). Consistently, we also found that after PM exposure activates NF-κB, the expression levels of cytokines and chemokines genes were up-regulated, and testicular inflammatory was increased. The potentiated testicular inflammation enlarged the empty areas of seminiferous tubules and decreased sperm count and normal morphology rate as previously reported in rat (14). Of note, another study reported that exposure to PM_2.5_ in mice did not increase testicular expression of *Il6* and *Tnfa* nor the rate of abnormal sperm (15). This inconsistency could be explained by the different dosages and origins of PM used in the studies, which requires further examination.

Activation of the DNA sensor cGAS requires a deacetylation step, and compounds that acetylate cGAS can suppress the cGAS-cGAMP-STING axis. Accumulating evidence has demonstrated the important role of mtDNA released from damaged mitochondria. Released mtDNA activate the innate immune system through several adaptors and may further mediate the testicular damage and inflammation (44). Here, we found that PM exposure induced mitochondrial injury and mtDNA release, causing testicular inflammation *via* cGAS-STING pathway. The findings were further established in cGAS knockdown TM3 cells. Importantly, PM induced orchitis and reduced offspring growth were alleviated by aspirin administration. The inflammatory response is a protective physiological program that protects the host against invading substances and pathogens. We believe that the best resolution of PM-induced inflammation is a process that allows for inflamed tissues, including the testes, to return to homeostasis. From this point of view, aspirin is a much better preventive choice than PDTC for the melioration of PM-induced orchitis due to its safety and confined effects (45). Under orchitis conditions, more immune cells including macrophages and dendritic cells can invade the testicular tubules and induce testis injury (16, 17). Inflammatory cytokines and chemokines are related to immune cells activation and recruitment in the testes (38). Inhalation of PM induces pulmonary inflammation, leading to the release of inflammatory mediators. Even though small amounts of particles might reach the testes, the pulmonary evoked systemic inflammation is thought to be the major factor to affect the testes. We found that aspirin-induced acetylation of cGAS inhibited the systemic inflammation in mice after PM exposure, especially in the testes. The reduced inflammatory factors in the testes, in turn, limited the immune cell invasion and alleviated orchitis. Consistently, we found increased invasion of neutrophils, dendritic cells, and macrophages in the testes of mice after PM exposure but reduced immune cell invasion after aspirin pre-treatment. Taking aspirin is considered a critical therapy for cardiovascular disease, and clearly aspirin treatment has side effects. We have preliminarily evaluated the hemorrhagic side effect in aspirin treated mice and found the risk of bleeding was increased from day 11 post aspirin administration. Together, the results indicated that both the benefits and side effects of aspirin intake to protect from PM induced orchitis need to be fully clarified further.

In summary, our present study provides compelling evidence that PM-induced orchitis in mice may be amenable to aspirin pre-treatment by acetylating cGAS. Surprisingly, we also found that aspirin administration in PM-exposed mice restored the growth of their offspring. It appears to provide an experiment-based view of the potential use of aspirin in the precaution of PM induced orchitis. However, despite our results, the mechanism underlying PM exposure and orchitis and the association between air pollutants and male infertility are not fully elucidated. Considering the complexity of ambient air pollution and the testicular immune regulatory system, further studies are required to clarify the mechanisms involved in infertility caused by environmental pollution.

## Data Availability Statement

The datasets presented in this study can be found in online repositories. The names of the repository/repositories and accession number(s) can be found below: NCBI SRA BioProject, accession no: PRJNA771215.

## Ethics Statement

The animal study was reviewed and approved by Kunming Institute of Zoology, Chinese Academy of Sciences Animal Care and Use Committee (SMKX-2019031).

## Author Contributions

TZ, XC, HQ, YL, JM, LZ, and LJ conducted the experiments. LJ and RL designed the experiments. WD provided guidance for the research. TZ and LJ analyzed the data and wrote the paper. All authors contributed to the article and approved the submitted version.

## Funding

This work was supported by the National Natural Science Foundation of China (NSFC) Grant (31900331, 32070444), Science and Technology Department of Yunnan Province (202001AW070019), Chinese Academy of Sciences “Light of West China” program and Youth Innovation Promotion Association (2019378) to LJ and funding from the NSFC (31930015), the CAS (XDB31000000, SAJC202103 and KFJ-PTXM-28), and Yunnan Province (2019-YT-053, 202002AA100007 and 2019ZF003) to RL.

## Conflict of Interest

The authors declare that the research was conducted in the absence of any commercial or financial relationships that could be construed as a potential conflict of interest.

## Publisher’s Note

All claims expressed in this article are solely those of the authors and do not necessarily represent those of their affiliated organizations, or those of the publisher, the editors and the reviewers. Any product that may be evaluated in this article, or claim that may be made by its manufacturer, is not guaranteed or endorsed by the publisher.
