# Supplementary material for: Aspirin Alleviates Particulate Matter Induced Asymptomatic Orchitis of Mice via Suppression of cGAS-STING Signaling
Source: Front Immunol. 2021 Dec 1;12:734546. doi: 10.3389/fimmu.2021.734546 (PMC8673441; doi:10.3389/fimmu.2021.734546)
Supplement: Supplementary file 1 [file DataSheet_1.pdf]

Supplementary Table 1. Primers used in this study.

Gene	5' primer	3'primer
Ifnb	GCCTTTGCCATCCAAGAGATGC	ACACTGTCTGCTGGTGGAGTTC
Cxcl9	GAGGCACGATCCACTACAAA	AGTCCGGATCTAGGCAGGTT
Tnfa	ATGAGAGGGAGGCCATTTG	CAGCCTCTTCTCATTCCTGC
Mcp1	CTTCTGGGCCTGCTGTTCA	CCAGCCTACTCATTGGGATCA
Il6	TGGTACTCCAGAAGACCAGAGG	AACGATGATGCACTTGCAGA
Cxcl1	ATGGCTGGGATTCACCTC	CTTCAGGGTCAAGGCAAG
Cxcl10	GCAGGTACAGCGTACGGTTC	CAGCAGAGGAACCTCCAGTC
Hprt	CTCATGGACTGATTATGGACAGGAC	GCAGGTCAGCAAAGAACTTATAGCC

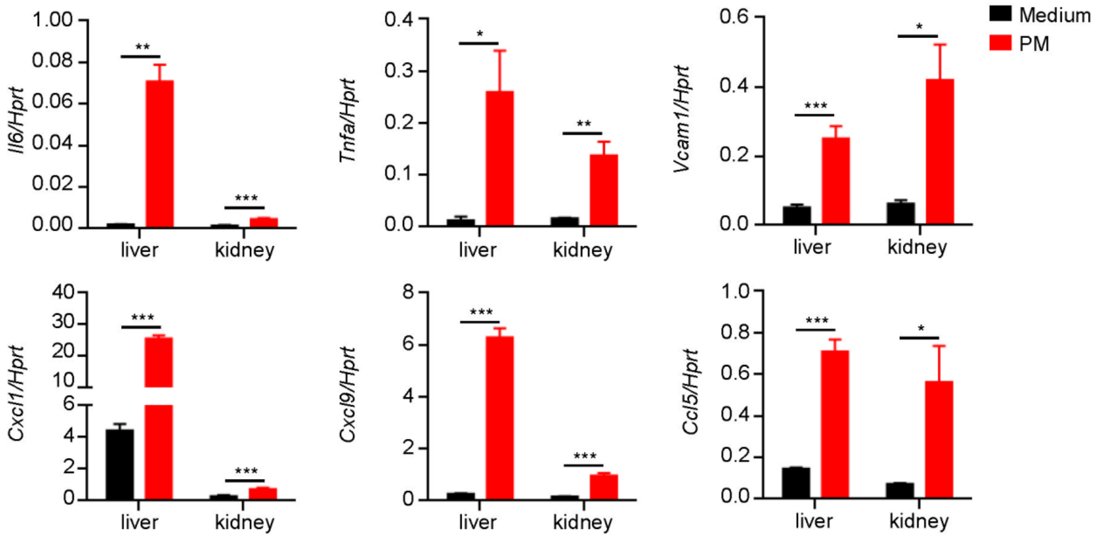

Supplementary Figure 1. Exposure of PM causes inflammation in liver and kidney of mice. Expression analysis of *Il6*, *Tnfa*, *Vcam1*, *Cxcl1*, *Cxcl9* and *Ccl5* in livers and kidneys after 7-days of PM exposure. Data represent two independent experiments. Data are presented as mean ± SEM. *, $P < 0.05$; **, $P < 0.01$; ***, $P < 0.001$.

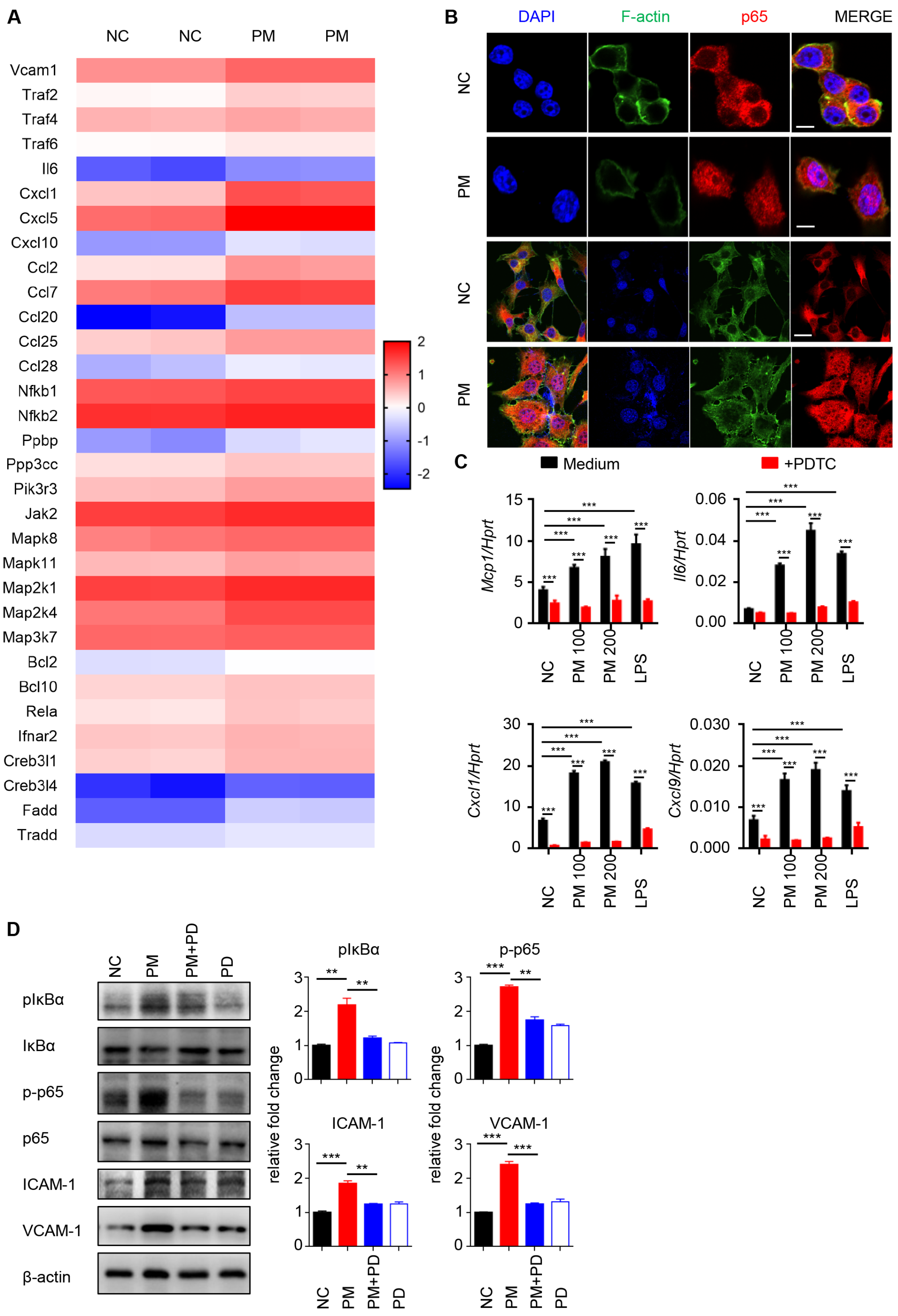

Supplementary Figure 2. PM activates NF-κB signaling in mouse Leydig cells. (A)

Heatmap showing the expression of genes comprised in NFκB signaling after 200

μg/ml PM administration for 24 hours. **(B)** Confocal microscopy analysis of p65 in TM3 cells after 200 μg/ml PM administration for 12 hours. Scale bars: 50 μm in yellow and 20 μm in white. **(C)** Expression analysis of *Mcp1*, *Il6*, *Cxcl1* and *Cxcl9* in TM3 cells after PM administration for 12 hours (NC, PBS; PM 100, PM at 100 μg/ml; PM 200, PM at 200 μg/ml; LPS, 1 μg/ml). **(D)** Immunoblot analysis of phosphorylation of IκBα and p65 and protein level of IκBα, VCAM-1, ICAM-1 and p65 in mouse testis after 3 day's of exposure. β-actin was used as the loading control. The intensity of signaling was quantified. Data represent three independent experiments in **B-C**. Data are presented as mean ± SEM. **, $P < 0.01$; ***, $P < 0.001$.

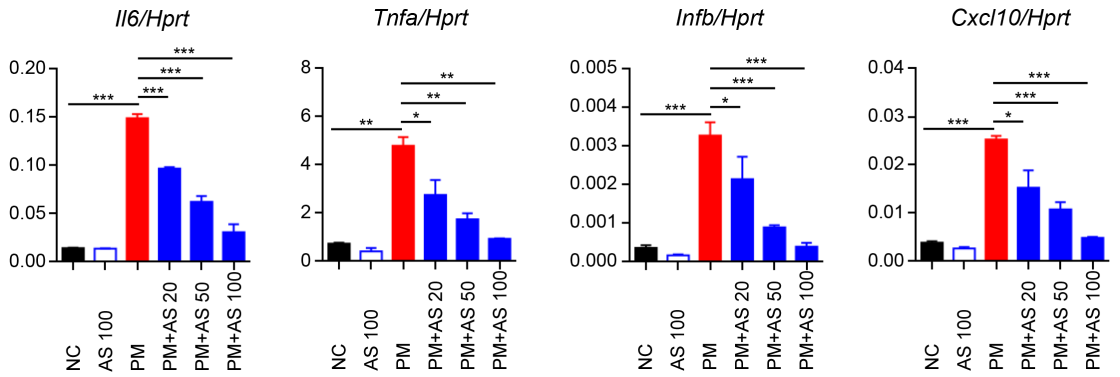

Supplementary Figure 3. aspirin pre-treatment reduces PM exposure induced inflammation (related to Figure 3). Expression analysis of *Il6*, *Tnfa*, *Ifnb,* and *Cxcl10* in the testis TM3 cells post 200 μg/ml PM administration for 12 hours with or without 20, 50 or 100 μg/ml aspirin. Data are presented as mean ± SEM. *, $P < 0.05$; **, $P < 0.01$; ***, $P < 0.001$.

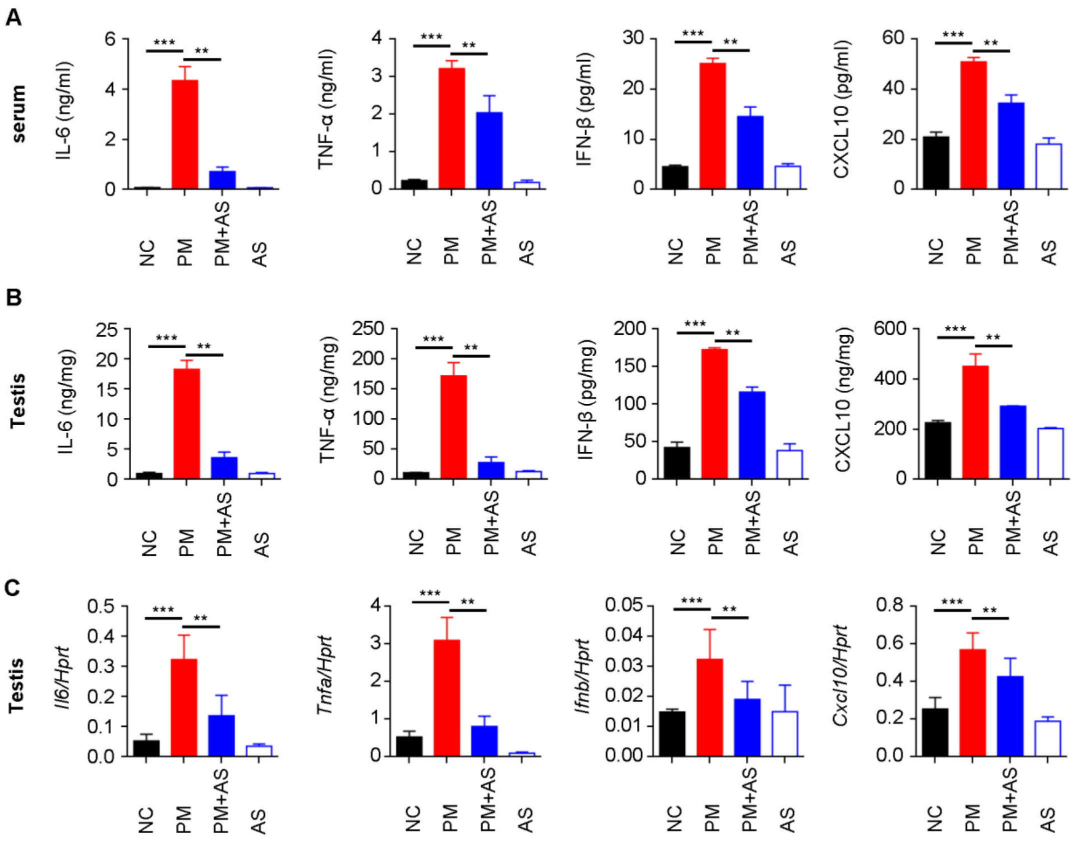

Supplementary Figure 4. Aspirin pre-treatment alleviates PM-induced systemic and testicular inflammation in mice. (A) Production of IL-6, TNF-α, IFN-β and CXCL10 in the serum of mice after 7-days of PM exposure with or without intragastrically administration of aspirin. (NC, PBS; PM, 400 μg/kg PM; AS, aspirin 20 mg/kg only; PM+AS, 400 μg/kg PM + 20 mg/kg aspirin). **(B)** Production of IL-6, TNF-α, IFN-β and CXCL10 in the testes of mice after 7-days of PM exposure with or withoutintragastrically administration of aspirin. **(C)** Expression analysis of *Il6*, *Tnfa*, *Ifnb*, and *Cxcl10* in the testes of mice after 7-days of PM exposure with or without intragastrically administration of aspirin. Data are presented as mean ± SEM. **, $P < 0.01$; ***, $P < 0.001$.

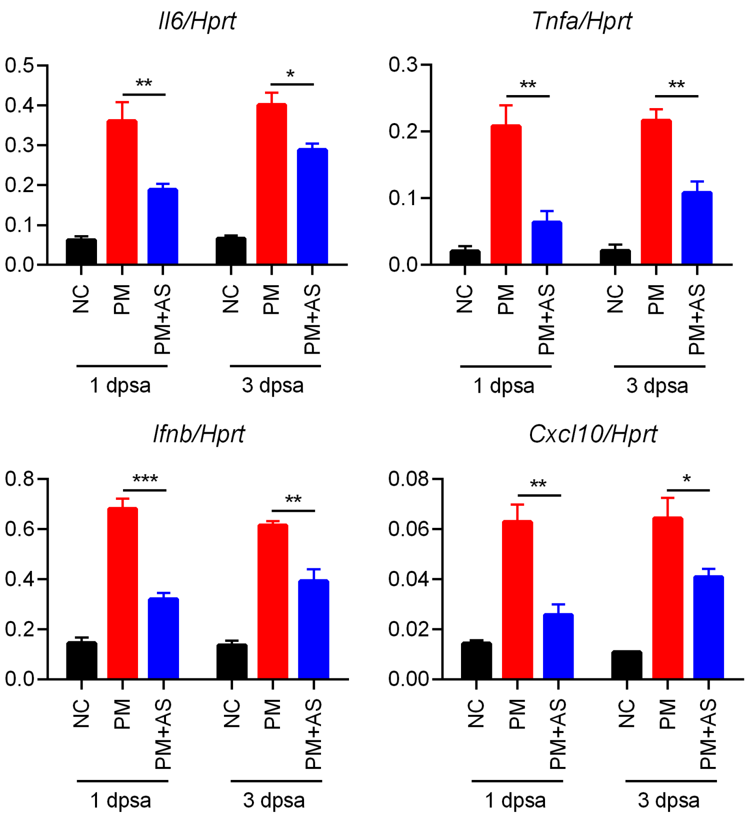

Supplementary Figure 5. The retained protective effect of aspirin. Expression analysis of *Il6*, *Tnfa*, *Ifnb*, and *Cxcl10* in the testes of mice after 200 μg/ml PM administration at indicated time. Mice were intragastrically administration of 20 mg/kg aspirin for 5 days before PM exposure. dpsa, days post stopping asipirin administration. *, $P < 0.05$; **, $P < 0.01$; ***, $P < 0.001$.

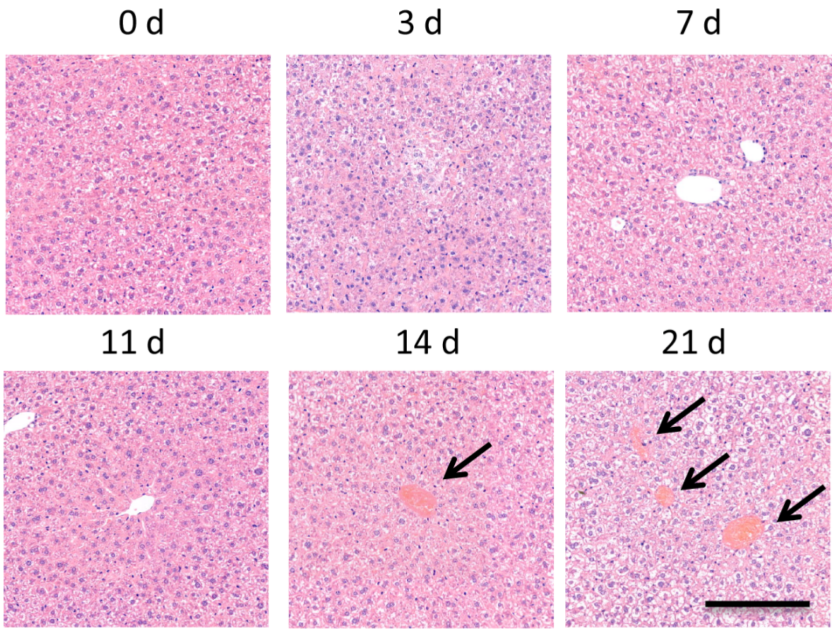

Supplementary Figure 6. Preliminary evaluation of hemorrhagic side effect of aspirin treated mice. H&E staining evaluation of the hemorrhagic spot (black arrow) in the livers of mice taking 20 mg/kg of aspirin for indicated time.
