# Supplementary figures and images for: Aspirin Alleviates Particulate Matter Induced Asymptomatic Orchitis of Mice via Suppression of cGAS-STING Signaling
Source: Front Immunol. 2021 Dec 1;12:734546. doi: 10.3389/fimmu.2021.734546 (PMC8673441; doi:10.3389/fimmu.2021.734546)

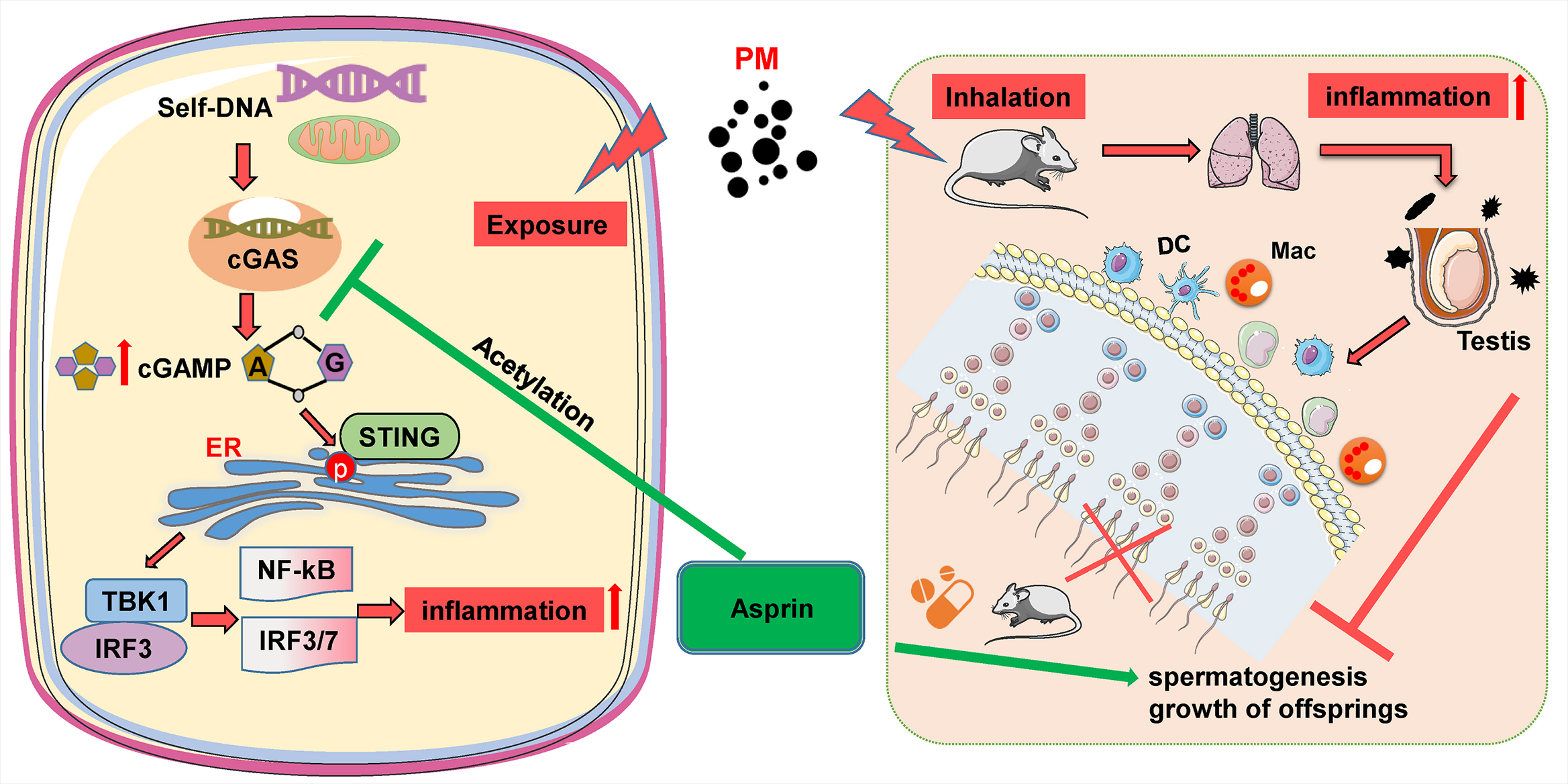

Supplement: Supplementary file 2 [file Image_1.tiff]
